# Exploring the interpersonal-, organization-, and system-level factors that influence the implementation and use of an innovation-synoptic reporting-in cancer care

**DOI:** 10.1186/1748-5908-7-12

**Published:** 2012-03-01

**Authors:** Robin Urquhart, Geoffrey A Porter, Eva Grunfeld, Joan Sargeant

**Affiliations:** 1 Cancer Outcomes Research Program, Cancer Care Nova Scotia, Victoria Building, QEII Health Sciences Center, 1276 South Park Street, Halifax, Nova Scotia, Canada; 2 Interdisciplinary PhD Program, Faculty of Graduate Studies, Dalhousie University, Henry Hicks Academic Administration Building, 6299 South Street, Halifax, Nova Scotia, Canada; 3 Department of Surgery, Faculty of Medicine, Dalhousie University, Victoria Building, QEII Health Sciences Center, 1276 South Park Street, Halifax, Nova Scotia, Canada; 4 Department of Community Health and Epidemiology, Dalhousie University, Centre for Clinical Research, 5790 University Avenue, Halifax, Nova Scotia, Canada; 5 Ontario Institute for Cancer Research, MaRS Centre, 101 College Street, Toronto, Ontario, Canada; 6 Department of Family and Community Medicine, University of Toronto, 500 University Avenue, Toronto, Ontario, Canada; 7 Division of Medical Education, Faculty of Medicine, Dalhousie University, Clinical Research Centre, 5849 University Avenue, Halifax, Nova Scotia, Canada; 8 Continuing Medical Education, Faculty of Medicine, Dalhousie University, Clinical Research Centre, 5849 University Avenue, Halifax, Nova Scotia, Canada

**Keywords:** Cancer, Synoptic report, Innovation, Implementation

## Abstract

**Background:**

The dominant method of reporting findings from diagnostic and surgical procedures is the narrative report. In cancer care, this report inconsistently provides the information required to understand the cancer and make informed patient care decisions. Another method of reporting, the synoptic report, captures specific data items in a structured manner and contains only items critical for patient care. Research demonstrates that synoptic reports vastly improve the quality of reporting. However, synoptic reporting represents a complex innovation in cancer care, with implementation and use requiring fundamental shifts in physician behaviour and practice, and support from the organization and larger system. The objective of this study is to examine the key interpersonal, organizational, and system-level factors that influence the implementation and use of synoptic reporting in cancer care.

**Methods:**

This study involves three initiatives in Nova Scotia, Canada, that have implemented synoptic reporting within their departments/programs. Case study methodology will be used to study these initiatives (the cases) in-depth, explore which factors were barriers or facilitators of implementation and use, examine relationships amongst factors, and uncover which factors appear to be similar and distinct across cases. The cases were selected as they converge and differ with respect to factors that are likely to influence the implementation and use of an innovation in practice. Data will be collected through in-depth interviews, document analysis, observation of training sessions, and examination/use of the synoptic reporting tools. An audit will be performed to determine/quantify use. Analysis will involve production of a case record/history for each case, in-depth analysis of each case, and cross-case analysis, where findings will be compared and contrasted across cases to develop theoretically informed, generalisable knowledge that can be applied to other settings/contexts. Ethical approval was granted for this study.

**Discussion:**

This study will contribute to our knowledge base on the multi-level factors, and the relationships amongst factors in specific contexts, that influence implementation and use of innovations such as synoptic reporting in healthcare. Such knowledge is critical to improving our understanding of implementation processes in clinical settings, and to helping researchers, clinicians, and managers/administrators develop and implement ways to more effectively integrate innovations into routine clinical care.

## Background

Cancer treatment and management have become increasingly complex over the past two decades, with therapeutic decisions often based on input from a multidisciplinary team that consists of radiologists, surgeons, pathologists, and oncologists [1]. For patients with suspected or confirmed cancer, clear and thorough recording of diagnostic and surgical procedures and findings support accurate diagnosis and staging. Such recording also facilitates more accurate prognosis estimates, post-operative management, and adjuvant treatment planning. The dominant method of reporting findings from diagnostic tests/procedures, surgery, and pathology examinations is the narrative report, which is a free text, descriptive account of the procedure, suspected or confirmed findings, and proposed treatment. Physicians dictate this report, often through automated telephone systems, and professional transcriptionists transcribe the oral description into a written document that is eventually placed into a patient's medical record. Research has demonstrated that narrative reports inconsistently provide the information required to understand the disease and make informed patient care decisions [2-7].

Another method of reporting, the synoptic report, captures data items in a structured manner and contains only items critical for understanding the disease and subsequent impacts on patient care. There is a spectrum of what is generally considered a synoptic report [1], from synoptic-like structured templates without scientifically validated elements to sophisticated electronic systems with drop-down menus, discrete data fields, standardized language, automated coding processes, and strong evidentiary basis. A landmark study in the early 1990s, which audited pathology practice patterns at 532 institutions in three countries, found that the one practice associated with completeness of pathology reporting for colorectal cancer specimens was use of a standardized report or checklist [8]. Since that time, researchers have consistently demonstrated that synoptic reports (even paper-based 'checklist' formats) vastly improve the quality of pathology reporting in colorectal [1,2,9-13], breast [1,9,14-16], lung [1,17], prostate [1], pancreatic [18], melanoma [19], and hematolymphoid cancers [20]. More recently, synoptic reporting has been shown to improve the quality of surgical reporting for a variety of malignancies, including colorectal [7], breast [21], thyroid [22], and pancreatic cancers [23], as well as non-malignant operative procedures [24,25].

Electronic synoptic reporting tools also lead to health system efficiencies compared to the dominant, dictated method of reporting [25-27]. Laflamme *et al. *[25] showed that use of synoptic templates accelerated the mean time for a verified surgical report to reach the patient's medical record by 800-fold compared to narrative reporting (28 minutes versus > 14 days, respectively). Moreover, the mean time from the end of the surgery to initiating the report was substantially less when using synoptic templates (0.43 hours) versus dictation (9.7 hours). Similar efficiencies were demonstrated in subsequent studies [26,27]. In a Canadian study, for example, 97% of synoptic reports were finalized, placed in the patient's medical record, and sent to all health professionals involved in the patient's care within 24 hours of surgery compared to a mean of 90 days for narrative reports [26]. Researchers have also estimated considerable cost-savings through the elimination of transcription services [25,26].

Beyond improving completeness of reporting and availability/immediacy of reports, synoptic reporting tools have the potential to improve quality of care by integrating practice guidelines/best evidence into report templates [21,26] and providing an efficient, real-time mechanism to generate data from the diagnostic and peri-operative periods [21,26,28,29]. These data may be used to provide real-time performance feedback to physicians and surgeons as well as enable improved process and outcomes measurement. International jurisdictions are increasingly endorsing synoptic reporting, including actively supporting/funding the implementation of synoptic templates [30-32] and providing commendation status to pathology labs that include a synoptic synopsis of scientifically validated data elements in their reports [33]. In addition, the professional pathology colleges in Canada, US, UK, and Australia have formalized a collaboration to develop common, internationally agreed-upon, standardized cancer reporting protocols [34].

The synoptic report represents a complex innovation (*i.e*., new knowledge, tool, or practice) in cancer care, with its implementation and use requiring fundamental shifts in physician behaviour and practice culture [35] as well as support from the organization (*e.g*., changes in institutional policies/processes) and larger system (*e.g*., governance arrangements, integration with health information technology infrastructure). Despite the demonstrated benefits, some physicians have reported reluctance to use synoptic reporting tools, with concerns including lack of flexibility in reporting complex procedures/cases [36,37], the prospect of being monitored [36], and discomfort with using information technology [37,38]. Changing physician reporting practice is a complex undertaking that requires comprehensive approaches at different levels of the health system [39]. This may be particularly true for narrative reporting, a practice that has existed for millennia [40].

### Implementing new practices in healthcare organizations

Knowledge translation (KT) research has largely focused on potentially useful strategies (*e.g*., opinion leaders, academic detailing, reminder systems) for improving the adoption and uptake of evidence (*e.g*., clinical practice guidelines) into practice [41]. Most of these strategies fall within the realm of individual-level interventions [39,42-44], with the target being 'autonomous' clinicians who are deemed to be more-or-less independent in their capacity to assemble and apply knowledge to modify their practices [41]. Despite the sizable amount of literature in this area, however, numerous systematic reviews have been unable to demonstrate which of these strategies work best, or even consistently, across clinical settings [43-45]. Many researchers have emphasized the unpredictable, slow, and haphazard nature of research implementation and use processes, with interventions working some of the time in some situations, but not at other times in seemingly similar situations [41,42,46], and the reasons for these differences unclear [47].

In reality, many organizational and socio-political (*e.g*., inter-organizational networks, funding arrangements) factors affect whether individuals in clinical settings actually make changes in their practice [41,47-50]. Much research has demonstrated the importance of organizational characteristics (*e.g*., culture, leadership, management support, evaluation/feedback mechanisms, and presence of champions) to implementation efforts in healthcare settings [41,51-65]. Moreover, many of the defining features of healthcare systems, including the range and diversity of stakeholders, complex governance/resource arrangements, and professional autonomy and specialization of many of its staff, result in many different cultures and norms as well as high levels of interdependency amongst professionals in the system [66,67].

Consequently, many implementation processes in healthcare organizations will also be characterized by a high degree of interdependency amongst organizational members [68,69]. Indeed, many innovations introduced in healthcare will require coordinated use by many individuals and professional groups to achieve benefits (electronic medical records are one example). These individuals are situated in organizational relationships wherein the implementation and use of a new tool or practice will ultimately be influenced by many interpersonal processes, including 'coalition building,' rhetoric, and persuasion [70,71]. Thus, while individual-level interventions are important to change clinical practice, the complex nature of healthcare organizations means individual-level interventions alone cannot change clinical practice in a widespread, sustainable way [39,48,72-75].

Understanding the dynamics of innovations in organizations has a long history in management and organizational sciences [76]. Rogers [77] has conceptualized the innovation-decision process as one that unfolds in distinct stages whereby an organization moves from initial awareness or knowledge of an innovation to eventually successfully integrating the innovation into ongoing processes (or, alternatively, rejecting the innovation). Contrary to this perspective, extensive longitudinal study of innovation processes led Van de Ven *et al. *[78] to describe the 'innovation journey' as a non-linear cycle during which ideas are developed (or adapted) and put into practice by people who, through their relationships and negotiations with others, make the changes necessary to implement the innovation within a specific organizational context. They highlight that people and relationships are instrumental to this journey, which is characterized by many divergent and convergent activities wherein the initial idea often leads to multiple ideas/actions, setbacks and delays occur frequently, staff experience high levels of elation and frustration, notions of success change, and new interdependencies are established that affect the wider organization. This broader 'systems' perspective [78,79] has recently made its way into KT dialogue and debate [49], challenging our thinking of a linear view of KT (*e.g*., researcher-push model) and moving us toward one that is much more contextual, relational, and 'living' in nature.

### Research objective

The objective of this study is to examine the key interpersonal, organizational, and system-level factors (hereafter referred to as 'multi-level' factors) that influence the implementation and use of synoptic reporting in three specific cases of cancer care. The interpersonal level relates to the relational aspects at the level of the implementation team/program: *e.g*., teamwork and team dynamics, communication, partner engagement, coalition building, power dynamics, and use of rhetoric and persuasion to accomplish goals/tasks. The organizational level relates to institutional (*i.e*., hospital) factors that influence implementation and behaviour change: *e.g*., organizational culture, leadership, management, intra-organizational relationships, evaluation capacity/mechanisms, implementation policies and practices, infrastructure, and presence of champions. The system level refers to the broader sociopolitical context: *e.g*., policies such as financial incentives/disincentives, resource and governance arrangements, and inter-organizational norms and networks.

This study involves three initiatives (the cases) in Nova Scotia, Canada, that have implemented a synoptic reporting tool within their departments/programs. The examination of each case will involve answering the following specific research questions:

1. What, if any, common factors affected implementation and use across cases? How was it that these factors 'transcended' the different contexts (setting, timing, and 'actors' involved)?

2. Are there context-specific factors within each case, which were not found in other cases, that affected implementation and use? If so, what are they and what are their specific relationships to the setting, timing, and actors?

The outcome of this study will be a descriptive and explanatory account of the multi-level factors that influence the implementation and use of synoptic reporting in cancer care.

### Methodology

Case study methodology (CSM) [80,81] will be used to study the three synoptic reporting cases in-depth, explore which factors were barriers or facilitators of implementation and use, examine relationships amongst factors, and uncover which factors appear to be similar (and distinct) across cases. CSM permits the rigorous study of a contemporary phenomenon within its real-life context [81], and of the complex interactions between the social actors and their actions and environments [82]. Case studies typically focus on 'how' and 'why' questions and explore multiple dimensions of some particular phenomenon. Flyvbjerg [83] argues that such in-depth study (of real cases in specific contexts) may be pivotal to transitioning from a novice to an expert understanding of the phenomenon.

This complexity means that case study researchers deal with distinct contexts whereby there are more variables of interest than data points. As a result, case studies rely on multiple sources of evidence and benefit from knowledge of the literature and existing theoretical perspectives [81]. The use of multiple sources is vital to CSM, as it permits corroboration (*i.e*., triangulation) of findings and resultant interpretations [81]. The use of existing theoretical perspectives helps guide data collection and analysis. Without a prior theoretical understanding, researchers risk spending considerable time and effort gathering basic information and 'providing description without meaning' (Hartley, cited in [84]).

## Methods

This research will examine the implementation and use of synoptic reporting tools for cancer care in Nova Scotia, Canada, using an explanatory multiple-case design. Explanatory case studies present data to explain how and why events happened; the researcher interprets phenomena by answering questions of how and why drawing upon a theoretical basis [81].

### Theoretical perspectives

The use of theoretical frameworks/perspectives provides structure to the inquiry and analysis, and helps ensure the findings from the case study are connected to and informed by the broader body of literature. This research is informed by the empirical and theoretical literature on research implementation and the diffusion/management of innovations. In particular, three theoretical frameworks/perspectives have largely informed the design of this study (see Table 1): Promoting Action on Research Implementation in Health Services [50,60]; Organizational framework of innovation implementation [59]; and 'Systems' thinking/change [49]

**Table 1:** Description of the three theoretical perspectives guiding the case study

Construct/Factor	Description
Promoting Action on Research Implementation in Health Services (PARiHS)	

Evidence	'[K]nowledge derived from a variety of sources that has been subjected to testing and has found to be credible' [85]. Four sources of evidence are research, clinical experience, patient experience, and local information.

Context	The 'environment or setting in which people receive healthcare services, or... the environment or setting in which the proposed change is to be implemented' [86]. Context consists of:

	• Culture manifests itself through the values, beliefs, and assumptions embedded in organizations and is reflected in 'the way things are done around here' [86].

	• Leadership 'summarizes the nature of human relationships such that effective leadership gives rise to clear roles, effective teamwork, and effective organizational structures' [86].

	• Evaluation includes performance monitoring and feedback at the individual, team, and system levels.

Facilitation	A 'technique by which one person makes things easier for others' [60]. Facilitation models range from doing for others to enabling others.

Organizational framework of innovation implementation	

Management support	Managers' commitment to the implementation process, including investments in quality implementation policies and practices.

Financial resource availability	The actual or potential resources that allow an organization or team adapt to, implement, and sustain change.

Implementation policies and practices	'[T]he formal strategies (i.e., the policies) the organization uses to put the innovation into use and the actions that follow from those strategies (i.e., the practices)' [59].

Implementation climate	'Employees' shared perceptions of the importance of innovation implementation within the organization' [87]. The extent to which employees view innovation use is 'rewarded, supported, and expected within their organization' [88].

Innovation-values fit	'[T]he perceived fit between the innovation and professional or organizational values, competencies and mission' [59].

Champions	'Charismatic individuals with significant personal authority who identify with the innovation and throw their weight behind its adoption and implementation' [59].

The need for systems change*	

Nature of knowledge	'The way in which participants (individuals) in the system understand the nature and characteristics of the new piece of knowledge and accept it' [49].

Local autonomy	The extent to which individuals, team, and the unit involved 'can make informed, autonomous decisions about how they can use the new knowledge to improve outcomes' [49].

(Re)Negotiation	How individuals 'negotiate and renegotiate relations with others (individuals, teams, internal, external relations) in their system' [49].

Resources	How individuals 'attract necessary resources to sustain the changes/improvements in practice' [49]. Involvement of key stakeholders at various levels of the system is critical to controlling and attracting resources.

*In this recent theoretical paper, Kitson [49] critiqued the critical social science, action science, diffusion of innovations, practice development, management of innovations, and learning organizations and systems theories literature to explore the underlying assumptions and theories used to describe healthcare systems and how knowledge is translated into practice.

Importantly, these perspectives were not identified with the aim of determining which is 'best' at explaining implementation and use processes in the cases selected for study. Rather, these perspectives, when taken together, present a range of interpersonal, organizational, and system influences on practice change and thus identify potentially important factors to study.

### Sampling

In case study research, limiting one's study to three or four cases will help ensure that a researcher is able to study each case in sufficient detail and depth [80,89]. In this study, three cases will be studied: Synoptic reporting in the Nova Scotia Breast Screening Program (NSBSP); Synoptic reporting in the Colon Cancer Prevention Program (CCPP); and Synoptic reporting in the Surgical Synoptic Reporting Tools Project (SSRTP).

These cases have been sampled on the basis of replication logic [81] as well as Stake's criteria [80]: relevance to the phenomenon; provision of diversity; and provision of good learning opportunities. Using replication logic, these cases were selected as they converge and differ with respect to factors that, based on the literature and theoretical perspectives, are likely to influence the implementation and use of an innovation in clinical practice. For example, the implementation of all three initiatives has involved formal leadership, relatively small implementation teams, clinical champions, and the development of monitoring and feedback mechanisms. At the same time, the cases represent diverse contexts, including differences in relevant professional groups (*e.g*., specialties, disease sites), institutions (*e.g*., academic/tertiary care centres, community hospitals), mode of change (*e.g*., top-down, bottom-up), implementation support and resource characteristics, and history/timing.

### Data collection procedures

This study will use multiple data collection procedures, gathering evidence across cases as well as across the various levels (interpersonal, organization, system) of each case, to gain rich, detailed information about each case and to increase the likelihood of achieving triangulation of data.

### Interviews with key informants

One-on-one semi-structured interviews will be conducted with key informants at the different levels of each case. For each case, a minimum of 14 to 16 key informants will be interviewed (see Table 2): users of the synoptic reporting system (*e.g*., radiologists, gastroenterologists, surgeons); individuals directly involved in planning or carrying out the implementation; organizational members relevant to the initiative; individuals involved at the system level (*e.g*., funders, policy-makers); and users of the final synoptic report (*e.g*., oncologists, coders). While the latter group may not have been directly involved in implementation efforts, their acceptance and use of the synoptic report is important to widespread implementation and use. Some informants may be asked to partake in several interviews (*e.g*., initial and follow-up interviews) depending on the case and data collected.

**Table 2:** Proposed key informants

CASE*	DESCRIPTION OF INFORMANTS^†^
NSBSP	• 4-5 radiologists
	
	• 3-4 implementation personnel (leaders, team members)^‡^
	
	• 3 organizational members (*e.g*., managers/directors of relevant departments)
	
	• 2 executive- or funding-level decision-makers
	
	• 2 report end-users (*e.g*., surgeons, coders)

CCPP	• 4-5 gastroenterologists/general surgeons
	
	• 3-4 implementation personnel (leaders, team members)^‡^
	
	• 3 organizational members (*e.g*., managers/directors of relevant departments)
	
	• 2 executive- or funding-level decision-makers
	
	• 2 report end-users (*e.g*., surgeons, radiation oncologists, coders)

SSRTP	• 4-5 surgeons
	
	• 3-4 implementation personnel (leaders, team members)^‡^
	
	• 3 organizational members (*e.g*., managers/directors of relevant departments)
	
	• 2 executive- or funding-level decision-makers
	
	• 2 report end-users (*e.g*., radiation oncologists, coders)

**Minimum number of key informants = 42-48**	

*NSBSP = Nova Scotia Breast Screening Program; CCPP = Colon Cancer Prevention Program; SSRTP = Surgical Synoptic Reporting Tools Project.

^**†**^The specified number represents the minimal number of key informants per category.

^‡^Implementation personnel may be interviewed on several occasions (*e.g*., initial and follow-up interviews) depending on the case and data collected.

Patton [90] and Rubin and Rubin [91] will be used to guide the interview design and research questions. Interview questions will be adapted based on each case's unique context as well as the person being interviewed and his/her role in the implementation. The semi-structured format will permit the interviewer to remain focused so that the research goals are achieved and the participant's time is used efficiently, yet also provide the freedom to probe additional issues that may be pertinent to the current research, but are not specifically addressed by the interview script [90]. Following each interview, the questions and responses will be reviewed to determine whether or not the issues were answered in sufficient depth and, if not, questions will be revised before the next interview [91]. Though theoretical perspectives have been used to guide this study, when information arises that conflicts with these perspectives, we will depart from the interview script and explore that particular concept/issue further. In subsequent interviews, that issue will be integrated into the script, if relevant in the context of that specific informant.

One investigator (RU) will conduct all interviews. Each interview will be audiotaped to ensure the data are retrievable and captured in true form, and will be transcribed verbatim by an experienced research coordinator.

### Non-participant observation

Non-participant observation [90] will be utilized to observe training sessions (format, quality of training) and initial surgeon reactions to viewing/using the innovation. Thus, these sessions will provide another opportunity to collect data on surgeons' perspectives on the innovation and any barriers that surgeons perceive at the time of training. These sessions will be conducted for one case only (SSRTP) since the implementation of the surgical synoptic report is ongoing, permitting prospective observation of user training and early support activities.

### Document analysis

Document information will be sought out and analyzed for each case. This includes project plans, team/organizational records related to synoptic reporting, training/support manuals, agendas and meeting minutes, formal/informal evaluations conducted, and media or professional articles/newsletters on initiative. These records will be reviewed to gain an historical and contextual perspective on the initiative and to corroborate and augment evidence from both interviews and observations [81]. Where documentary evidence conflicts with findings from other sources, we will attempt to resolve these contradictions through further inquiry (*e.g*., follow-up with informants, contact with implementation team).

### Physical artifacts

Each synoptic reporting tool will be examined to gain insight into the technical operations related to using the system. This will entail inputting 'test' cases into the system to experience tool use as well as viewing the final synoptic report to observe its design/format. Field notes/perceptions related to these experiences will be used to corroborate and augment evidence (specifically related to system/tool issues) from other sources.

### Tool audits

Tool audits will be conducted to determine the proportion of eligible clinicians using the synoptic reporting tool and the proportion of eligible procedures at each institution that were reported using the synoptic reporting tool. This will entail an audit of the synoptic reporting system/database as well as the relevant institutional administrative system (*e.g*., admission/discharge/transfer or operating room scheduling systems). The latter is required to determine the number of eligible procedures (*e.g*., endoscopies, surgeries) performed at that institution in a specified period of time. Eligibility criteria for the audits include the physician/surgeon is a registered user on the synoptic reporting system and a synoptic template is in use for the specific procedure (*e.g*., lumpectomy for a malignant breast tumour).

### Analysis

Yin [81] describes a number of important strategies of case study analysis: developing case descriptions, relying on theoretical frameworks/perspectives, using data from multiple sources to augment and triangulate findings, and examining rival explanations (*i.e*., other plausible explanations for the findings; one rival explanation is that psychological theories, such as the Theory of Planned Behaviour [92], better explain implementation in one or more of the cases studied). In this study, data analysis will involve a three-stage process: production of a case record/history for each case; in-depth analysis of each case; and cross-case analysis. Like other qualitative methodologies, analysis will begin with the first data collected.

The first stage in the analytic process involves case description. That is, a detailed case record (or history) will be constructed for each case, including an in-depth description of the history and context of the initiative (including the impetus for the initiative, timeline, key milestones and activities, and organization of the project and implementation). This descriptive record will also involve situating the case within its socio-political context, particularly as it pertains to the provincial healthcare environment at the time of implementation.

The second stage will attempt to gain an in-depth understanding of each synoptic reporting initiative and how its experiences relate to the research objective as well as the theoretical literature. This stage will involve four analytic steps, which will be performed separately for each of the three cases:

1. Thematic analysis for each of the following evidence sources: interviews, documentary evidence, and observation. This analysis will follow the thematic analysis approach presented by Braun and Clarke [93], involving coding, collating codes, and generating, reviewing, and refining themes. This approach is similar to the analysis steps outlined by other researchers [89,91,94]. NVivo 9 (QSR International, Australia) will be used to help manage the data and aid in coding processes.

2. Cross-source analysis of themes. This analytic process will compare, contrast, and synthesize findings from each source to gain an understanding of how the data from each source corroborates and augments data from other sources, and to identify any areas of inconsistency and potential contradiction.

3. Explanation building to integrate evidence, link the data to theoretical perspectives/literature, and develop a deeper understanding of what occurred [81]. This technique involves iteratively and flexibly moving back and forth between prior knowledge (theoretical perspectives, other literature, research objective) and emerging, case-specific data to get a deeper understanding and theoretically-sound explanation of what actually happened and what was important throughout the process. An important aspect of this process is considering and questioning other explanations. This process will enable us to explain the implementation processes and the multi-level factors that influenced implementation and use in each case, and to examine existing theoretical constructs and determine their appropriateness to these contexts. In this way, we are able to explore existing theoretical perspectives and revise theory, when appropriate.

4. Presentation of findings in relation to the overall objective of the study, the theoretical perspectives, and rival explanations.

The final stage will be to conduct a cross-case analysis to compare and contrast themes between the cases. Each case will be treated as a separate study and findings (similarities and differences) will be compared across cases to develop theoretically informed, generalisable knowledge on implementing innovations in clinical practice that can be applied to other settings and contexts [81].

One critique of CSM is that case studies are subject to confirmation bias, specifically toward confirmation of preconceived ideas [83,95]. That is, researchers can selectively describe and explain the studied events 'to support a favoured theory by underplaying evidence inconsistent with the theory or supporting an alternative' (p. 164) [95]. To minimize confirmation bias in this study, all members of the research team will participate in components of the analysis and compare their findings. The focus will be to attend to all the evidence collected, display and present the evidence and interpretation(s) separately, consider other plausible interpretations, and seek out additional evidence where inconsistencies or contradictions exist [81]. Moreover, the research team will strive to increase the 'trustworthiness' of this study through detailed documentation and description, including development and maintenance of a case study database (consisting of a complete set of all the data collected, along with the treatment of the data during the research process), maintenance of a chain of evidence (or audit trail), and rich descriptions of each case and its context.

## Discussion

Well-conducted case studies can make significant contributions to the knowledge base of a particular area [96] and have the potential to transform practice, either within the case(s) being studied or across similar situations where individuals can learn from the findings [97]. Beyond informing the adoption and implementation of synoptic reporting, we anticipate this study will add to the development and application of theoretical knowledge (particularly 'systems' perspectives) in the growing KT field, and contribute to our knowledge base on the multi-level factors, and the relationships amongst factors in specific contexts, that influence implementation and use of innovations in healthcare organizations. Both contributions are important to improving our understanding of implementation processes in clinical settings, and helping researchers, clinicians, and managers/administrators develop and implement ways to more effectively integrate innovations into routine clinical care. This is especially relevant in the present healthcare environment wherein new knowledge and technologies are growing and changing rapidly, and the treatment and management of many diseases are increasingly complex and multidisciplinary.

## Abbreviations

CCPP: Colon Cancer Prevention Program; CSM: Case study methodology; KT: Knowledge translation; NSBSP: Nova Scotia Breast Screening Program; SSRTP: Surgical Synoptic Reporting Tools Project.

## Competing interests

GAP is the Project Lead in Nova Scotia for the Surgical Synoptic Reporting Tools Project. He receives no financial compensation for this work. The remaining authors have no conflicts of interest to declare.

## Authors' contributions

RU conceived the idea for this study, led the intellectual development and protocol writing, and will be primarily responsible for its conduct. GAP, EG, and JS all contributed to the drafting and development of the study. All authors reviewed and agreed on the final manuscript.
